# A New Methodology for Selecting CT Scanning Parameters Depending on the Density of Materials

**DOI:** 10.3390/ma17246172

**Published:** 2024-12-17

**Authors:** Ksenia Ostrowska, Jerzy Sładek, Paweł Wołkanowski, Ireneusz Dominik, Danuta Owczarek, Marek Nykiel, Krzysztof Tomczyk, Michał Stoliński

**Affiliations:** 1 Mechanical Faculty, Cracow University of Technology, Jana Pawła II 37 avenue, 31-864 Krakow, Poland; ksenia.ostrowska@pk.edu.pl (K.O.); jerzy.sladek@pk.edu.pl (J.S.); pawel.wolkanowski@pk.edu.pl (P.W.); danuta.owczarek@pk.edu.pl (D.O.); stolinski.michal@gmail.com (M.S.); 2 Faculty of Mechanical Engineering and Robotics, AGH University of Krakow, al. Adama Mickiewicza 30, 30-059 Krakow, Poland; dominik@agh.edu.pl; 3 Faculty of Materials Engineering and Physics, Cracow University of Technology, Jana Pawła II 37 avenue, 31-864 Krakow, Poland; marek.nykiel@pk.edu.pl; 4 Faculty of Electrical and Computer Engineering, Cracow University of Technology, Warszawska 24, 31-155 Krakow, Poland

**Keywords:** X-ray computed tomography, material density, measurement accuracy, coordinate measurement, linear radiation absorption coefficient

## Abstract

The CT (computed tomography) scanner has been used for many years now not only for medical measurements but also in many industries, for example, in defectoscopy for measuring sheet thickness and checking the joining of materials, as well as for measuring the geometry of individual components. This type of scanner is a good complement to coordinate contact and non-contact measurements for intra-structural measurements and inaccessible places. The variety of materials, however, makes it very difficult to select individual CT parameters. In this paper, a curve for selecting the maximum and minimum voltage of the lamp depending on the density of a given material is determined and an interpolation polynomial (1d with a third-degree polynomial) is used, by defining third-degree glued functions (cubic spline) to determine intermediate voltage values to a given material density, so as to determine full data ranges. This approach can facilitate the work of selecting scanning parameters for non-destructive testing, as this is a difficult process and sometimes consumes half of the measurement time. The practical experiments were carried out at the Accredited Coordinate Metrology Laboratory to develop a multi-criteria matrix for selecting CT measurement parameters for measurement accuracy. This approach reduced the time by an average of half an hour and effectively optimized the selection of scanning parameters.

## 1. Introduction

Industrial measurement using a computed tomography (CT) system is an advanced technique that uses X-rays to scan an object and create a three-dimensional reconstruction of it. This technology is widely used in industry, especially in quality control and materials analysis [1,2,3]. The design is different from a medical measurement system, but the principle of measurement remains the same. This measurement is used for precise geometric measurements, internal defect analysis, quality control, reverse engineering, or testing of materials and composite components [4,5]. The use of CT capabilities for industrial testing is not fully recognized yet. It is involves a combination of non-nesting testing and typical coordinate metrology. Many works have examined the use of an application and comparison of CT to machines or systems to identify material defects, porosity, or material inclusions [6,7]. These tasks, though performed in different sectors, have one common conclusion: the measurement of internal defects using CT has one undeniable advantage, and that is the measurement range (the ability to measure large objects). As a result of this, on most occasions, we do not need to destroy the component to be measured; in the majority of cases, we are able to measure it in its entirety. There is also no need to prepare samples, scrap, or coatings to allow measurement. Thanks to this undoubted advantage, industrial CT has also found application in the analysis of recycled materials, such as waste cement, glass dust, and straw fibers, for example, where the effect of their content on the permeability of residual granite soils has been studied [8,9].

Although CT has long been used for medical measurements, it is only from about 2005 that CT has found its way into a geometry measurement system [10,11]. Unfortunately, for the measurement of industrial components, where manufactured parts must be measured with an accuracy class of 5–9, setting the appropriate parameters of CT is difficult and contains numerous parameters that can significantly change the process [12,13]. Parameter handling today poses a lot of problems, especially as most users in industry are not trained in physics, but rather in metrology and quality. Unfortunately, it is still the selection of parameters that changes the accuracy of a geometry measurement [14,15]. If the operator is inexperienced, the individual is not able to maintain the repeatability of the measurement, and the initial work of setting the appropriate parameters takes a significant part of the time spent on changeover from one component to another. A major influence that persists to this day remains unresolved—the measurement of components made of different materials—as it is only through the correct configuration of input data in the CT that we are able to achieve a good distinction between materials [16]. In industry, setting the right settings in CT is often done by trial and error. This is followed by measurement, which, with an average of 1500–2000 images from which a 3D model is assembled, varies between 0.5 h on the so-called ‘fast’ setting and up to 4 h on the ‘precise’ setting. In order to address these challenges, an input selection matrix will be developed at the Accredited Coordinate Metrology Laboratory, validated by accurate measurements on CMMs. In this paper, one of the elements of the matrix will be presented, enriched by an interpolation method based on third-degree glued functions (cubic spline), where the spline method uses functions defined as low-degree polynomials separately for each segment between adjacent interpolation nodes.

**Figure 1:**
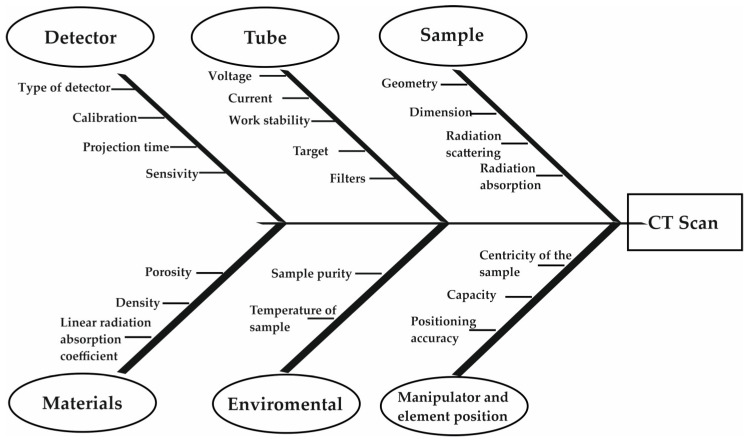
Elements affecting the accuracy of the measurement of the CT performed [11].

Research into improving the accuracy and suitability of CT for use in various technical fields has been ongoing for many years. As Figure 1 shows, there are many distortions and elements that affect the application of the CT for subsequent tasks [17]. The first problem, which is still not fully resolved and divides the user and manufacturer community, is the calibration and sometimes the adjustment of these devices. Manufacturers have developed standards that adjust the machine within a small range of the measurement space, which users disagree with, repeatedly proving that the position of the measuring element in different parts of the space is crucial. This is at least related to the resolution of the system [18,19]. Another important aspect on which research was conducted was the analysis of the impact of algorithms for assembling 2D images into a 3D object. In medical research, it has been found that a reconstructive iterative algorithm improves qualitative analysis, as the amount of noise increases with decreasing thickness and at cross-sections [20].

Subsequent research has been conducted on CT artifact reduction methods, which have also introduced ambiguity in the results. In [21], a new method was proposed to reduce CT artifacts by using multiple X-ray CT scanning, in which CT data fusion is combined with optimized selection of scan angle combinations. In [22], a projection sinogram-based artifact correction method was proposed. The proposed technique calibrates geometric deviations in an industrial computed tomography (CT) system with parallel angles and effectively eliminates geometric artifacts in reconstructed CT images. In [23], a modification of a single-grid phase contrast X-ray imaging (PCXI) system was proposed using a Fourier domain analysis technique to extract absorption, scattering, and differential phase contrast images. The proposed modification involves rotating the X-ray grating on the image plane to achieve spectral separation between the desired information and the moiré stripe artifact, which is introduced by the superpositioning of the periodic grating shadow image and periodic sampling by the detector. This optimization was intended to increase the spectral separation between the fundamental spectrum (lower frequency) and the spectral harmonics (higher frequency) used to extract different image contrasts. 

An extension of the research presented above is the work on the introduction of virtual optimization solutions to reduce time or improve measurement accuracy [24,25]. In [26,27], meanwhile, the impact of spectrum pre-filtering and beam hardening correction on internal and external dimensional measurements is evaluated using a proven simulation tool. Similar research, but supported by methods using deep learning algorithms that process data in both the image domain and frequency space, which increases the efficiency of artifact correction, is described by the authors in [28,29]. In [30,31], it is described what industrial users find difficult in evaluating the uncertainty of CT measurements and the metrological performance of CT systems. This paper examines the state-of-the-art systems in industrial CT metrology, with a focus on accuracy and traceability issues, by examining specific results obtained from the first international comparison of CT systems for dimensional metrology. The comparison included 15 CT systems operated by experts in Europe, America, and Asia.

**Figure 2:**
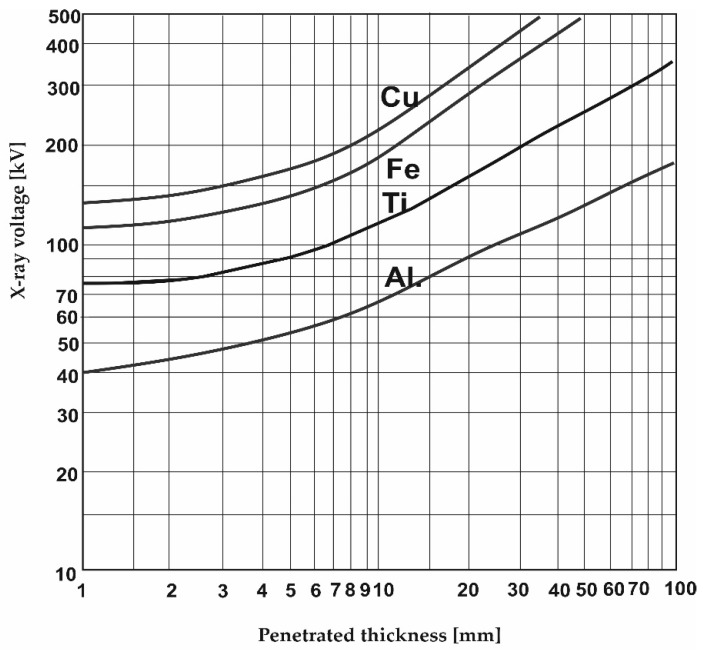
Relationship between the selected voltage and material thickness [25].

As presented above, CT used for industrial measurements is still not fully recognized. When working with CT, a major problem is the selection of scanning parameters [32,33]. There are some input data upon which to base the type of plot of the maximum voltage set on the X-ray tube against the thickness of several basic materials like Cu, Fe, Ti, or Al (Figure 2).

However, this is not a sufficient basis, especially if CT is used in small batch production, where we have constant changes of products as well as their materials. Searching constantly for new parameters for each job causes too much downtime, which companies cannot afford [34]. Therefore, in this article, samples were designed with a known density that increased logarithmically. Initially, one material was used, where the density of the material was increased by compression. Unfortunately, the resulting parameters had too small a variation (from 6.139 to 7.037 $g/{cm}^{3}$) for any noticeable differences to be observed in the choice of scanning parameters for screening the material. Samples with a large amplitude of variation in material density (from 0.661 to 8.429 $g/cm^{3}$) were then made because the same material in terms of atomic composition can have different densities (e.g., liquid and vapor water) [1,16,28]. For the measurement, the manufacturer’s settings, at which device adjustments are performed, were used. During the tests, samples of the same volume and dimensions were used, but with increasing density and, consequently, mass. A plot of the minimum and maximum voltage set on the tomography lamp against the density of the material was determined. Polynomial interpolation (1d with a third-degree polynomial) was used, by defining third-degree glued functions (cubic spline) to determine intermediate voltage values up to a given material density, so as to determine full data ranges. Each sample’s dimensions were measured on a Coordinate Measuring Machine at the Accredited Coordinate Metrology Laboratory of the Cracow University of Technology to serve as a denominator for the research. 

## 2. Measuring Geometry with CT

**Figure 3:**
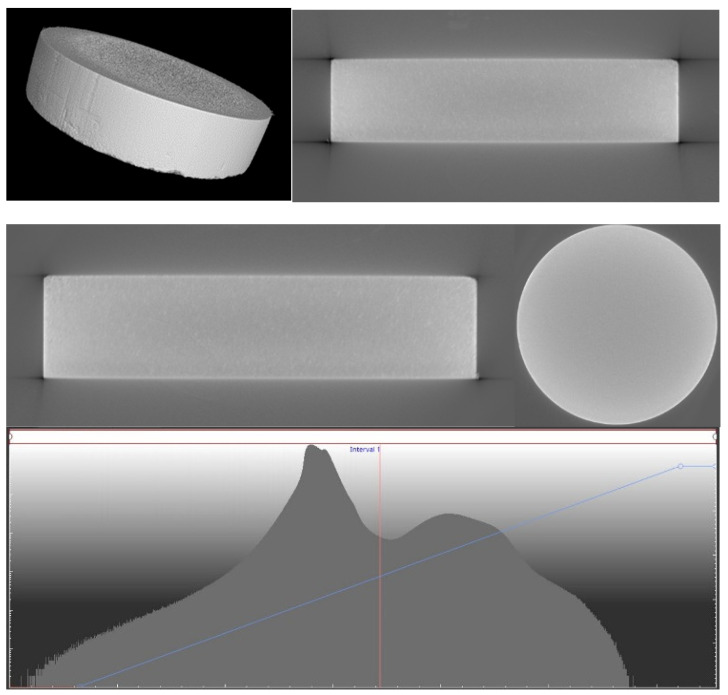
The 3D model after reconstruction; cross-section from the front after reconstruction; and cross-section from the side after reconstruction.

The X-ray computed tomography provides imaging based on X-rays of the object under examination. The radiation beam, as it passes through the object, is attenuated—a process that depends on the thickness of the absorbing material, the absorption coefficient referenced, or the density of the material to units of the same length. During the measurement process, hundreds or thousands of 2D X-ray images are usually taken for different angular positions of the lamp-detector system relative to the object being measured [34]. As a result of the reconstruction from the 2D shots, a 3D spatial image is obtained (Figure 3).

The X-ray tomography is classified as a non-destructive test. X-rays are characterized by their ability to penetrate bodies that are in different states of aggregation, and, when they pass through a material, they are weakened. This relationship can be described by a linear absorption coefficient, where a given medium of a certain thickness transmits the same fraction of the number of photons; the coefficient number is shown in Equation (1):

(1) $$N_{x}=N_{0}e^{\mu g}$$

where $N_{x}$—number of photons after passing through the medium, $N_{0}$—number of incident photons, μ—linear absorption coefficient of radiation, and g—thickness of the sample.

The intensity of X-rays depends on the number of photons—Equation (1), and this can also be described by the dependence of the intensity of radiation after passing through the object ($I_{x}$) in relation to its initial value ($I_{0}$), as follows:

(2) $$I_{x}={I_{0}}^{\mu g}$$

where $I_{x}$—intensity of radiation after passing through the medium, $I_{0}$—initial value of radiation intensity, and μ—linear absorption coefficient of radiation.

As can be seen from Equation (2), the permeability depends on factors such as the thickness of the absorbing material (g) and the absorption coefficient (μ) related to the same units of length. The linear absorption coefficient of radiation depends on the wavelength and atomic number of the material being permeated (3):

(3) $$\mu =k\lambda^{3}Z^{3}$$

where μ—linear absorption coefficient of radiation, k—proportionality factor, λ—wavelength of radiation, and Z—atomic number of the material of the medium being scanned.

An increase in the atomic number of the material of the medium weakens the penetrability of X-rays. The linear absorption coefficient can be replaced by the mass coefficient, which expresses the probability of interaction of radiation with a unit mass of material, and is given by:

(4) $$\mu_{m}=\mu /\rho$$

where $\mu_{m}$—mass absorption coefficient, μ—linear radiation absorption coefficient, and ρ—material density of the medium.

## 3. Experimental

### 3.1. Material Preparation and Testing

In order to determine the selection of setting parameters depending on the density of the material, 15 samples with densities ranging from 0.661 $g/{cm}^{3}$ to 8.429 $g/{cm}^{3}$ were measured; where the last value did not give positive results, the measurements could not be made without changing the rest of the device settings (Table 1).

The material that was used to make the samples was a mixture of powders—one powder was based on aluminum and the other on leaded bronze and Acrawax lubricant. These powders were unilaterally pressed in a rigid die using different pressures, and the weights were prepared based on a compressibility curve developed in-house. Sintering was carried out by heating to an isothermal sintering temperature at a rate of 10 °C/min, annealing at 900 °C for 30 min, and then cooling also at a rate of 10 °C/min. Sintering was carried out in a 5.0 nitrogen atmosphere, with a nitrogen flow rate of 80 mL/min. Samples marked with numbers had fixed proportions of ingredients and were pressed at different pressures in samples marked with letters; the samples also had different proportions of ingredients. In the presented publication, the idea was to prepare test materials that, with relatively constant dimensions, would present different densities. In order to obtain different densities without changing the chemical composition (only the proportions of the components change), samples produced by powder metallurgy methods were used. Such products produce different relative density and porosity as a function of parameters such as pressing pressure or sintering temperature. Powders were used which, after sintering, produced a similar structure. The bases of the cylindrical specimens were sanded successively on 120, 240, and 600 water papers so that parallelism was maintained for the base surface and so that the measurement result was not significantly affected. 

Cylindrical samples of the tested materials were prepared on a TOP-300 universal lathe. Density tests were performed on a Pycnomatic ATC helium pycnometer from Thermo Scientific (Waltham, MA, USA) according to the ASTM D792 [35], ISO 1183 standard [36]. One of three measuring containers with a volume of 40 ${cm}^{3}$ was used, with a measurement temperature of 25 °C and a gas pressure stability of 0.001 kPa. A calibration method using certified stainless steel balls was performed. Repeatability and accuracy modes at the level of 0.01% were maintained. Density measurement accuracy was 0.001 $g/{cm}^{3}$. Helium 6.0 was used for the tests. Due to the quality of CT scans, the histogram was set in the range of 200–10,000.

The tests were performed on a Waygate Technologies Phoenix V|tome|x M Metrology Edition CT scanner (Cincinnati, OH, USA) with an accuracy of (3.8 + L/100 [mm]) μm, using a Microtube X-ray tube with a maximum power of 240 W with a measurement resolution of less than 1 μm. In addition, a Nanotube X-ray tube with a measurement resolution of 0.2 μm was installed in an industrial CT scanner located at the Coordinate Metrology Laboratory of the Faculty of Mechanical Engineering at the Cracow University of Technology. The 3D scanning measurement space was 420×400 mm.

**Figure 4:**
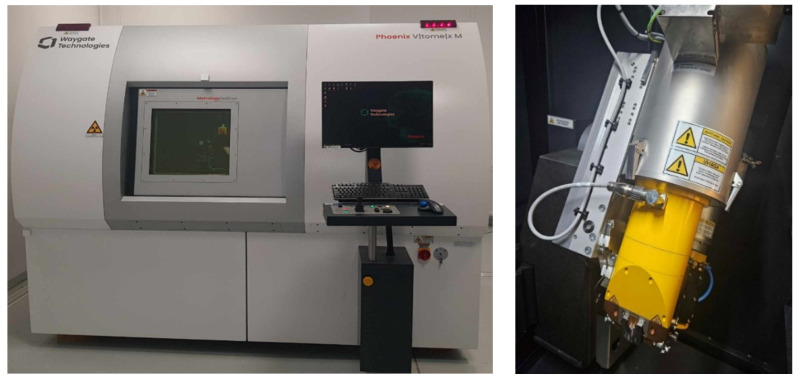
CT scanner and microtube.

Figure 4 shows the CT scanner and microtube.

The maximum weight of the sample can be as much as 50 kg. Table 2 includes the operating parameters of the tomography.

### 3.2. Calculation and Results

Voltage is responsible for the force with which photons fly through the material, and current is responsible for the photon flux density. Binning is responsible for combining pixels. Sensitivity affects the sensitivity of the detector by either enhancing or reducing it. This option is responsible for amplifying the signal and detecting small changes, but can lead to more artifacts. Timing is responsible for the detector’s exposure time to incoming radiation. The current value was as per the calibration of the device.

Each sample was placed in the CT space at the exact distance at which the manufacturer recommends performing the adjustment. This is very important, as the resolution of the measurement depends on the distance of the measuring element from the X-ray tube. Voltage adjustments, both minimum and maximum, were selected for each part. In this task, it should be noted that the device will indicate if the parameters are too high, providing a warning alarm about the possibility of damage to the device. The lower limit, on the other hand, is determined by checking the histogram readings and selecting them according to the manufacturer’s assumptions and based on the laws of physics.

The samples were measured according to the selected parameters, and their geometric parameters were then compared with the nominals mapped on the CMM. If the geometric parameters determined from the measurements obtained with the tomograph were within the accuracy limits specified by the manufacturer, it was considered that the parameters had been selected correctly. This was a very time-consuming task, as each change in lamp intensity resulted in, at minimum, another 20 min of measurement. Then, each time the correct values were selected, each measurement was performed 10 times to determine the measurement uncertainty.

**Figure 5:**
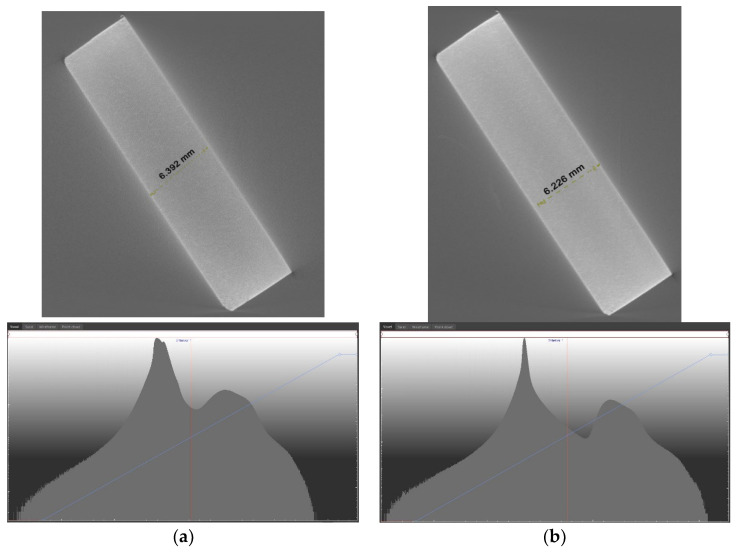
Scanned samples with an incorrect (**a**) and a correct (**b**) histogram.

From the results, a tube voltage range selection curve was determined for the given material densities. To show the errors when measuring with inappropriate parameters, the differences are shown in Figure 5.

**Figure 6:**
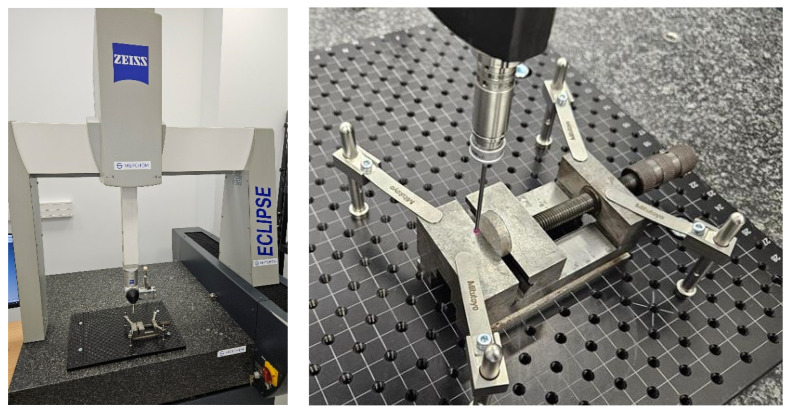
Measuring samples on a coordinate measuring machine Zeiss Eclipse (Oberkochen, Germany).

Figure 5a shows ring artifacts that were created during scanning. This is caused by improper tomography operating parameters, which caused the histogram not to be in the range of 200–10,000. On the other hand, in Figure 5b there are no ring artifacts because the scanning parameters were properly selected, and the histogram was in the range of 200–10,000. Results are from the selected sample—11. In the case of the scan from Figure 5a, the distance between the upper and lower planes was 6.392 mm, and, in the case of the sample from Figure 5b, the distance between the lower and upper planes was 6.226 mm. The height, as determined on the Zeiss Eclipse coordinate measuring machine, was 6.192 mm (Figure 6).

In all measurements, the more accurate results were from the appropriate range of the histogram, and the maximum difference was 6.2 μm.

**Figure 7:**
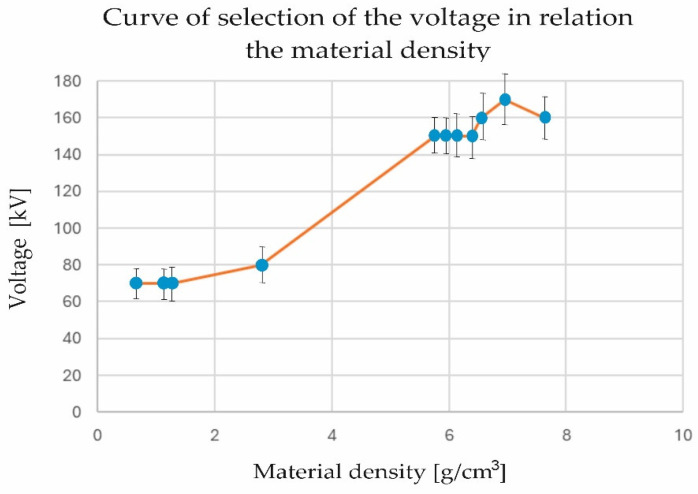
Curve of selection of the voltage in relation to material density.

Figure 7 shows the curve of selection of the voltage in relation to the material density.

As can be seen in Figure 6, as the density of the material increased, the voltage also increased; the graph indicates the non-linear nature of this relationship. For higher density values (from about 6 to 8 g/cm^3^), the voltage changes were smaller and more stable, and we even observed slight decreases, which may indicate a saturation point or limitation in the relationship. In summary, the graph illustrates that as the density of the material increased, the required voltage increased. However, after a certain point, the relationship became less intense, which could be an important consideration in the selection of process parameters.

In order to make the results complete, interpolations (1d with third-degree polynomials) were used to feed into the matrix for selecting the setting parameters from the material density by defining third-degree glued functions (cubic spline), also called (jargon-wise) splines. The spline method uses functions defined as low-degree polynomials separately for each segment between adjacent interpolation nodes. The presented local polynomials are selected in such a way that, in addition to the interpolation conditions, they satisfy the gluing conditions so that the whole spline is a function with sufficient regularity. In this case, there were 5 interpolation nodes (n+1) with coordinates $(x_{0},\;y_{0}),$ $(x_{1},\;y_{1}),\ldots$, $(x_{n},\;y_{n}),$ where x—material density and y—tension. It was necessary to find n polynomials of the third degree with equations connecting the points $(x_{i}-1,\;y_{i}-1),$ where i=1, 2, …, n, so that the line connecting them was smooth. Therefore, it was necessary to determine the values of 4*n* coefficients $a_{i}$, $b_{i}$, $c_{i}\;$, and $d_{i}$ for i=1, 2, …, n, as follows:

(5) $$f_{i}(x)=a_{i}+b_{i}x+c_{i}x^{2}+dx^{3}$$

For the task to be calculated correctly, certain conditions had to be formulated:

– using the assumption of continuity of the line, it was assumed that:
  (6) $$f_{i}(x_{i})=y,\;\;\;f_{i+1}(x_{i})=y_{i},\;\;\;\mathrm{for}\;\;\;i=1,\;2,\;\ldots ,\;n-1$$
– the first polynomial should pass through the initial point $\left(x_{0},\;y_{0}\right)$, while the last polynomial should pass through the final point $\left(x_{n},\;y_{n}\right)$, hence:
  (7) $$f_{1}(x_{0})=y_{0},\;\;\;f_{n}(x_{n})=y_{n},\;\;\;\mathrm{for}\;\;\;i=1,\;2,\;\ldots ,\;n-1$$
– it was also assumed that there is a condition at the interpolation nodes

(8) $${f\prime \prime }_{i}(x_{i})={f\prime \prime }_{i-1}(x_{i})$$

where i=1,2,…,n−1.

– Hence, the following was received:

(9) $$2c_{i}+6d_{i}x_{i}=2c_{i+1}+6d_{i+1}x_{i}$$

From the condition given by Equation (7), 2n−2 equations are obtained; from the conditions given by Equations (8) and (9), n−1 equation each, finally making 4n−2 equations. Two more equations are missing to solve the task. Therefore, in order to obtain splines with linear ends, it was assumed that:

(10) $${f\prime \prime }_{1}(x_{0})=0$$

which gives $2c_{i}+6d_{i}x_{0}=0,$ and

(11) $${f\prime \prime }_{1}(x_{n})=0$$

which gives $2c_{n}+6d_{n}x_{n}=0,$ while in order to obtain splines with parabolic ends, it was additionally adopted that:

(12) $${f\prime \prime }_{n}(x_{n})={f\prime \prime }_{n}(x_{n-1})$$

which gives $2c_{n}+6d_{n}x_{n}=2c_{n}+6d_{n}x_{n-1}$.

Finally, the second derivative for the ends of the interval were linearly extrapolated, as given by the following formulae:

(13) $$\frac{{f\prime \prime }_{1}(x_{1})-{f\prime \prime }_{1}(x_{0})}{x_{1}-x_{0}}=\frac{{f\prime \prime }_{2}(x_{2})-{f\prime \prime }_{2}(x_{2})}{x_{2}-x_{1}}$$

and

(14) $$\frac{{f\prime \prime }_{n}(x_{n})-{f\prime \prime }_{n}(x_{n-1})}{x_{n}-x_{n-1}}=\frac{{f\prime \prime }_{n-1}(x_{n-1})-{f\prime \prime }_{n-1}(x_{n-2})}{x_{n-1}-x_{n-2}}$$

in order to enter the splines with the ends of the third degree into the matrix.

Figure 7 shows the interpolation of the minimum lamp intensity adjusted to the material density.

**Figure 8:**
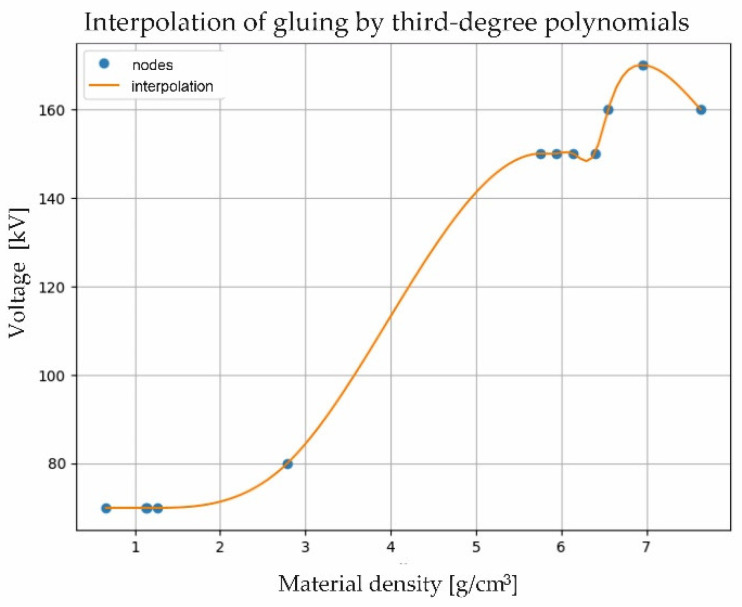
Interpolation of the minimum lamp intensity adjusted to the material density.

When using interpolation with third-degree glued functions (cubic spline) for different material densities and corresponding tensions, each spline segment is only responsible for interpolation between adjacent nodes, meaning that any change in node values (e.g., the addition or correction of a single point) only affects the local segment of the curve. This reduces error propagation and helps to keep the whole model stable. Third-degree splines provide a good approximation without the excessive oscillations that are typical of higher degree polynomial interpolation. This means that the correspondence between material density and voltage is reproduced precisely, which is beneficial when creating models based on empirical data. Through their use, it is possible to create tables or matrices for the selection of parameters in an accurate and homogeneous manner, which facilitates subsequent adjustment of system parameters to specific material density values. The use of third-degree spline interpolation to model the dependence of na-tension on material density allows this dependence to be reproduced accurately while maintaining curve smoothness. This is useful in the context of technical parameter modeling, where the voltage–density relationship must be represented continuously and without sudden changes in value (Figure 8).

Table 3 shows the voltage values (in kV) calculated by third-degree spline interpolation for different material densities (in g/cm^3^) in the gaps between the interpolation nodes.

These values were generated from third-degree spline functions. The generated values allow the presented relationship to be entered into a matrix, which is designed in the laboratory, for the selection of parameters for scanning with CT. Thanks to the use of third-degree interpolation, it was possible to model the influence of material density on the selection of lamp voltage over the entire range of capabilities of the CT scanner used for measurement. Materials with a density of more than 8.0 g/cm^3^ could no longer be X-rayed without the detriment of overheating the head. The software reported an alert about the risk of lamp damage. 

In order to verify the correctness of the presented method, 3 measurements each were made of 4 elements of similar thickness (to avoid the influence of material thickness), but with different densities from the interval where the parameters were interpolated. All measurements of geometric size were measured on a machine validating the size of the element, i.e., on a Zeiss Eclipse CMM, while density tests were performed on a Pycnomatic ATC helium pycnometer from Thermo Scientific according to ASTM D792, ISO 1183. The difference in the distance of the planes did not exceed 3.2 µm, which is within the limits of CT accuracy. 

**Figure 9:**
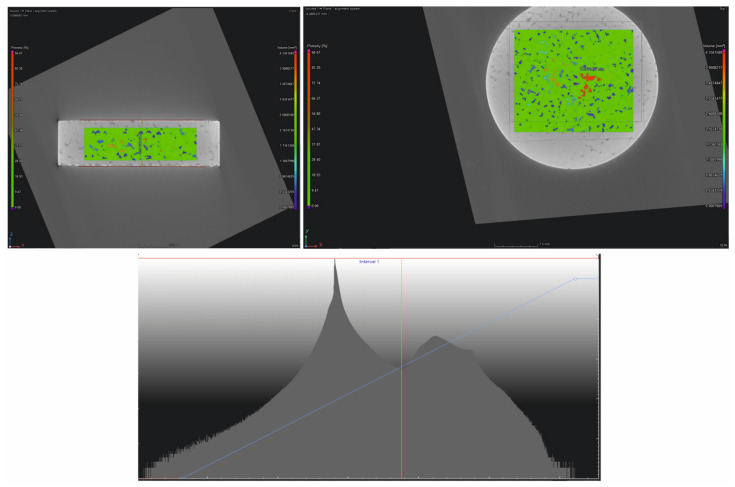
Follow-up measurement.

Figure 9 shows the measurement of a component with a thickness of 8.575 mm and a density of 4.621 g/cm^3^, along with the determination of porosity. The parameters were selected according to the values in Figure 8 and Table 3.

## 4. Conclusions

In this study, the influence of material densities on the selection of appropriate lamp parameters for CT measurement can be observed. A minimum voltage selection curve was determined for given material densities. The results ranged from 70 to 160 kV for densities from 0.661 to 7.632 $g/{cm}^{3}$. The maximum voltage remained at one level, i.e., 190 kV. In order to make the results complete, interpolations (1d with third-degree polynomials) were used to feed into the matrix for the selection of setting parameters from the material density, by determining the third-degree glued functions (cubic spline). Fifty tension values were generated, averaging every 0.200 $g/cm^{3}$ (Table 3 and Figure 8).

Work on the development of a multi-criteria matrix for the selection of CT measurement parameters in relation to the physical properties of materials is being carried out at the Accredited Coordinate Metrology Laboratory of the Cracow University of Technology. One of the parameters influencing the accuracy of the obtained measurement, as well as the appropriate selection of parameters for CT measurement, is the precise material density. In further work, analogous to the present study, curves will also be determined for the given material densities, but with a greater focus on increasing the thicknesses of the materials in question, as well as many other factors influencing the result of a CT measurement. 

A major challenge when it comes to the use of the computed tomography for metrology tasks is the measurement of components consisting of a minimum of two materials, i.e., electronic components and automotive components—for example, an entire lamp that additionally has a painted coating. This is a very problematic task because, when processing the results, the algorithm does not quite cope with the distinction between materials. Therefore, further work will be carried out that will also aim to make the ISO 50 algorithm, which is responsible for determining the boundary between one material and the other, dependent on parameters such as material density. The final stage of the research will be to work on the scalability of the results to more complex materials, multi-material components, composites, and materials with different structures. Research on the accuracy and mapping limit of individual materials and their structures is also planned.

## Figures and Tables

**Table 1:** Sample parameters.

Sample Number	Density D $\left[g/{cm}^{3}\right]$	Mass m [g]	Size V $\left[{cm}^{3}\right]$	Height h [mm]	Diameter d [mm]
C	0.661	2.472	3.739	7.956	24.463
G	1.126	3.448	3.061	8.030	22.031
F	1.136	3.548	3.123	7.887	22.455
D	1.266	4.549	3.592	7.900	24.061
A	2.785	8.718	3.130	7.877	22.493
10	5.749	17.838	3.103	6.071	25.510
11	5.934	18.448	3.109	6.078	25.520
12	6.139	19.147	3.119	6.064	25.591
13	6.392	19.928	3.117	6.079	25.553
14	6.551	20.310	3.100	6.050	25.543
15	6.638	20.627	3.107	6.061	25.549
16	6.866	21.239	3.093	6.043	25.530
17	6.957	21.330	3.066	5.987	25.535
B	7.632	23.289	3.052	8.019	22.012
E	8.429	27.407	3.252	7.779	23.070

**Table 2:** Operating parameters of the tomography.

Parameters	Value
Sensitivity	1
Binning	1 × 1
Current	300 μA
Voltage	70–220 kV
Timing	100 ms

**Table 3:** Results for 50 interpolation steps.

$\mathrm{Material}\;\mathrm{Density}\;[g/{cm}^{3}]$	Voltage [kV]	$\mathrm{Material}\;\mathrm{Density}\;[g/{cm}^{3}]$	Voltage [kV]	$\mathrm{Material}\;\mathrm{Density}\;[g/{cm}^{3}]$	Voltage [kV]	$\mathrm{Material}\;\mathrm{Density}\;[g/{cm}^{3}]$	Voltage [kV]	$\mathrm{Material}\;\mathrm{Density}\;[g/{cm}^{3}]$	Voltage [kV]
0.661	70	2.083	72	3.506	98	4.929	140	6.352	149
0.803	70	2.226	73	3.649	102	5.071	143	6.494	156
0.945	70	2.368	74	3.791	107	5.213	145	6.636	165
1.087	70	2.510	76	3.933	111	5.356	147	6.778	169
1.230	70	2.653	78	4.075	116	5.498	149	6.921	170
1.372	70	2.795	80	4.218	120	5.641	150	7.063	169
1.514	70	2.937	83	4.360	125	5.782	150	7.205	168
1.657	70	3.079	86	4.502	129	5.925	150	7.347	166
1.799	70	3.222	90	4.644	133	6.067	150	7.490	163
1.941	71	3.364	94	4.787	136	6.209	149	7.632	160

## Data Availability

The original contributions presented in this study are included in the article. Further inquiries can be directed to the corresponding author.
